# Impaired Carbohydrate Metabolism and Excess of Lipid Accumulation in Offspring of Hyperandrogenic Mice

**DOI:** 10.3390/metabo12121182

**Published:** 2022-11-26

**Authors:** Cynthia Aburto-Hernández, David Barrera, Rosario Ortiz-Hernández, Emilio Espinoza-Simón, Leticia Parra-Gámez, James González, M Luisa Escobar, Gerardo H Vázquez-Nin, Olga Echeverría-Martínez, Nayeli Torres-Ramírez

**Affiliations:** 1 Departamento de Biología Celular, Facultad de Ciencias, Universidad Nacional Autónoma de Mexico, Avenida Universidad 3000, Cd. Universitaria, Coyoacán, Mexico City 04510, Mexico; 2 Departamento de Biología de la Reproducción, Instituto Nacional de Ciencias Médicas y Nutrición Salvador Zubirán, Av. Vasco de Quiroga No. 15, Col. Belisario Domínguez, Sección XVI, Mexico City 14080, Mexico; 3 Departamento de Bioquímica y Biología Estructural, Instituto de Fisiología Celular, Universidad Nacional Autónoma de México. Avenida Universidad 3000, Cd. Universitaria, Coyoacán, Mexico City 04510, Mexico; 4 Departamento de Anatomía, Facultad de Medicina, Universidad Nacional Autónoma de México, Avenida Universidad 3000, Cd. Universitaria, Coyoacán, Mexico City 04510, Mexico

**Keywords:** insulin resistance, hyperandrogenism, fetal programming, hyperglycemia, maternal androgen excess

## Abstract

Polycystic ovary syndrome (PCOS) is an endocrine–metabolic disorder of unknown etiology. Hyperandrogenism (HA) is the main diagnostic criteria for PCOS, in addition to being a risk factor for developing several disorders throughout the patient’s life, including pregnancy. However, the impact on offspring is little known. Therefore, the aim of this work was to evaluate the effect of maternal HA on glucose metabolism and hepatic lipid accumulation in adult offspring. We used Balb/c mice treated with dehydroepiandrosterone (DHEA) for 20 consecutive days. The ovary of DHEA-treated mice showed hemorrhagic bodies, an increased number of atretic follicles, and greater expression of genes related to meiotic cell cycle and DNA repair. The DHEA offspring (O-DHEA) had low birth weight, and some pups showed malformations. However, O-DHEA individuals gained weight rapidly, and the differences between them and the control group became significantly greater in adulthood. Moreover, O-DHEA presented higher serum glucose after a 6 h fast and a larger area under glucose, insulin, and pyruvate tolerance test curves. Oil Red O staining showed a more significant accumulation of fat in the liver but no changes in serum cholesterol and triacylglycerol levels. In summary, our results show that HA, induced by DHEA, affects gene expression in oocyte, which in turn generates defects in embryonic development, insulin resistance, and alteration in hepatic gluconeogenesis and lipid metabolism in O-DHEA, thereby increasing the risk of developing metabolic diseases.

## 1. Introduction

The intrauterine environment is a critical element in the health of the progeny. During childhood and adulthood, it has been shown that undernutrition, overnutrition, and maternal glucocorticoid excess lead to adverse intrauterine conditions that induce long-term metabolism changes, thereby increasing the risk of neuroendocrine, metabolic, and psychiatric diseases [1,2].

Bennewitz in 1828 recorded the large size of the fetus born to a diabetic mother for the first time; years later, other studies supported this observation, suggesting a relationship between maternal hyperglycemia and metabolic alterations in newborns, including a higher percentage of subcutaneous fat, fetal hypoglycemia, and hyperinsulinism [3,4,5]. Thus, diabetes was considered as the conjunction of environmental factors and a thrifty genotype. This latter is helpful in conditions of food scarcity but hardly ever necessary today. Subsequently, Hales and Barker [6] proposed the thrifty phenotype hypothesis, in which suboptimal conditions in utero promote permanent adaptations during fetal development (fetal programming), which increases the probability of developing chronic noncommunicable diseases [7].

Polycystic ovary syndrome (PCOS) is an endocrine–metabolic disorder characterized by hyperandrogenism (HA) and ovulatory dysfunction. The most frequent metabolic alterations in patients with PCOS are visceral obesity, insulin resistance (IR), hyperinsulinemia, and type 2 diabetes (T2D), which contribute to HA and vice versa [8,9,10]. Of all these characteristics, HA ranks as one of the main factors that alter fetal programming [11]. The offspring of mothers with PCOS exhibit various effects on growth and development, including androgenization, metabolic, neuroendocrine, cardiovascular, and reproductive disturbances [12]. The PCOS fetal programming mechanism is associated with HA [11]. In sheep, prenatal overexposure to androgens increases insulin production due to an excess of beta cells in females [13]. Studies on daughters of women with PCOS demonstrated its association with body weight and body mass index, which increases the risk of obesity and T2D [14,15]. Sons of women with PCOS showed higher weight during early infancy and developed IR and metabolic syndrome during adult life [16,17,18]. However, more studies on the differences and similarities of the effects of PCOS according to gender are still needed.

It has been suggested that maternal PCOS creates epigenetic reprogramming in the embryo, associated with glucotoxicity, lipotoxicity, and inflammation [19]. Tarumi et al. [20] demonstrated oocyte degeneration in a PCOS mouse model with androstenedione, in addition to chromosomal misalignment and inhibition of spindle assembly and meiotic maturation affecting oocyte competence and function. Liu et al. [21] and Wood et al. [22] found that oocytes from PCOS patients have a distinct gene expression pattern, including genes related to the meiotic cell cycle, DNA repair, hormone receptor signaling, and gap junctions. Moreover, according to Wood et al. [22], genes overexpressed in PCOS oocytes have common sequence motifs with the androgen receptor and PPARγ, suggesting an altered metabolic environment. Here, we test the hypothesis that HA generates changes in maternal oocytes resulting in fetal reprogramming evidenced by metabolic disturbances in offspring. 

## 2. Materials and Methods

### 2.1. Animals

Female Balb/c mice were raised and housed under controlled conditions of light and temperature (12 h light, 12 h dark) with access to food and water ad libitum. All animal procedures were conducted pursuant to Official Mexican National Protection Laws of Animal Welfare NOM-062-ZOO-1999 and under the supervision of Sciences Faculty Commission of Academic Ethics and Scientific Responsibility (CEARC).

Twelve female prepubertal Balb/c mice (25 days old) were randomly divided into PCOS and control groups. The PCOS-like model was developed by subcutaneously injecting DHEA (Catalog No. D4000, Sigma; 6 mg/100 g body weight) dissolved in sesame oil once daily for 20 consecutive days, while the control group was injected with sesame oil without DHEA. The animals were weighed every two days during the entire treatment period. Daily vaginal wet smears determined the estrous cycle stage at the same time of day. Mice with irregular or loss of estrous cycles were considered PCOS-like models. After hormone treatment, ovaries from 6 mice (3 DHEA and 3 control) were dissected for paraffin embedding.

In order to evaluate the effect of maternal HA on pups, superovulation was induced in 6 mice by exogenous chorionic gonadotropin treatment (eCG/hCG). Totals of 10 IU each of equine chorionic gonadotropin (eCG) (Novormon^®^ 5000, Syntex, Argentina) and human chorionic gonadotropin (hCG) were intraperitoneally administered (Choriomon, IBSA, Institut Biochimique SA, Lugano, Switzerland) 48 h apart [23]. Subsequently, control males were used for mating purposes.

The pups born in the control group (O-control; 10 females, 8 males) and the group treated with DHEA (O-DHEA; 6 females, 7 males) were weighed every two days for three months, and the estrous cycle was evaluated in adult mice. In addition, glucose, insulin, and pyruvate tolerance tests were performed, and peripheral blood serum cholesterol and triglyceride levels were measured using a tail vein drip. Finally, the liver was dissected for Oil Red O staining.

### 2.2. Hormonal Assay Test

At the end of the treatments, blood samples from DHEA and control females were obtained by cardiac puncture and the serum was separated and stored at −20 °C until assayed. Progesterone, testosterone, and estradiol levels were quantified by a solid-phase competitive chemiluminescent enzyme assay (Immulite Catalog No. LKPW1, LKTW1, and LKE21, respectively; Siemens Healthcare Diagnostics Products Ltd., Los Angeles, CA, USA) on a Siemens Immulite 1000 analyzer (Siemens Healthcare Diagnostics Inc., Flanders, NJ, USA), pursuant to the manufacturer’s instructions.

### 2.3. Morphological Analysis and Ovarian Follicle Counting

Mice were euthanized with isoflurane 24 h after the last injection, and their ovaries were rapidly removed, carefully dissected, and fixed in 4% paraformaldehyde in phosphate-buffered saline (PBS) at pH 7.2 for 24 h at room temperature. Tissues were then dehydrated in gradual alcohol and embedded in paraffin. Finally, whole ovaries were serially cut into 5 µm sections.

For hematoxylin and eosin staining, sections were deparaffinized and rehydrated. After that, sections were stained with Gill’s hematoxylin solution for 10 min, incubated for 1 min in Scott solution, rinsed in tap water for 10 min, and then immersed in 30, 50, and 70% alcohol for 1 min. Next, samples were stained with alcoholic eosin for 3 min, dehydrated in gradual alcohol, and submerged in xylol for 5 min. Finally, the slides were covered with synthetic resin. Follicles were classified as primordial, primary, secondary, preantral, and antral. To ensure that follicles were not counted more than once, they were only counted when the nucleolus had been identified.

For periodic acid–Schiff (PAS) staining, sections were deparaffinized, rehydrated, incubated in 0.5% periodic acid for 10 min, rinsed in water, and incubated for 60 min in Schiff’s fuchsin-sulfite reagent (Catalog No. S5133, Sigma, Saint Louis, MO, USA). Next, slides were washed under running tap water, counterstained with hematoxylin, rinsed in Scott’s solution and water, dehydrated, cleared, and mounted using synthetic resin.

For argentic staining, sections were deparaffinized, rehydrated, and rinsed in ammonia water. Slides were immersed in ammoniacal silver carbonate solution for 3 h at 60 °C and rinsed in water. Finally, slides were incubated with 10% formol for 1 min, rinsed in water, dehydrated, cleared, and mounted using synthetic resin.

For Masson’s trichrome stain, ovaries were fixed in Bouin’s solution (Catalog No. 16045, Polyscience, Warrington, PA, USA), dehydrated in gradual alcohol, and embedded in paraffin. Next, ovary sections were deparaffinized, rehydrated, stained with Weigert’s iron hematoxylin for 10 min, rinsed in water, and incubated for 10 min in Biebrich Scarlet-Acid Fuchsin. Next, sections were incubated in 2.5% phosphotungstic–phosphomolybdic acid solution for 10 min and 2.5% Aniline Blue for 5 min. Finally, slides were rinsed in water, incubated in 1% acetic acid for 1 min, dehydrated, cleared, and mounted using synthetic resin.

### 2.4. Oil Red O Staining

Newborn and four-month-old O-control and O-DHEA individuals were euthanized with isoflurane and had their livers removed. The tissues were fixed in 4% paraformaldehyde in PBS at pH 7.2 during 24 h at room temperature and rinsed in tap water. Liver cryosections (8 µm) cut from snap-frozen OCT-embedded tissue blocks were incubated in freshly prepared 0.2% Oil Red O solution for 20 min and counterstained with hematoxylin for 4 min. Next, sections were incubated for 3 min, immersed in Scott’s solution to give the hematoxylin stain a bluish tint, and rinsed in tap water. Each slide was divided into ten frames at 20× magnification. The hepatic lipid droplets were semiquantified using the ImageJ software.

### 2.5. Glucose Tolerance Test

For glucose tolerance tests (GTT) in offspring, basal glucose was measured in 12 h fasting mice (time 0). Then, 15 min after an intraperitoneal injection of glucose (1.5 g/kg weight), tail blood was collected and glucose levels measured with Accu-Chek^®^ Performa (Roche, Mannheim, Germany. This was performed again to measure blood glucose values for 30, 60, 90, and 120 min after the glucose injection.

### 2.6. Insulin and Pyruvate Tolerance Test

For insulin tolerance tests (ITT) in offspring, basal glucose was measured in 6 h fasting mice (time 0). Then, 15 min after an intraperitoneal insulin injection (0.75 IU/kg weight), tail blood was collected, and glucose levels were measured with Accu-Chek^®^ Performa (Roche, Mannheim, Germany). This was performed again to measure blood glucose values for 30, 60, and 90 min after the glucose injection. For pyruvate tolerance tests (PTT), mice received 2 g/kg weight pyruvate intraperitoneal injection at 30, 45, 60, and 120 min [24].

### 2.7. Triacylglycerols and Cholesterol Measurement

Serum triacylglycerols and cholesterol were measured from the tail blood of overnight fasting offspring using Accutrend^®^ Plus (Roche, Mannheim, Germany).

### 2.8. Ovarian RNA Extraction

Total RNA was isolated from control and PCOS-like 45-day-old mice (6 control and 6 treated with DHEA) using TRIzol (Invitrogen) and purified by chloroform extraction and isopropanol precipitation. RNA integrity was verified by electrophoresis in a denaturing agarose gel. Total RNA was digested with DNAse I (Catalog No. D-4263, Sigma, Saint Louis, MO, USA) to remove any contaminating genomic DNA. cDNA synthesis reactions were performed using a First Strand cDNA Synthesis Kit (Catalog No. K1612, Thermo Scientific™, Vilnius, Lithuania).

### 2.9. Quantitative Real-Time PCR

Real-time PCR amplifications (qPCR) were performed using the standard curve method with specific primers for the Actb (ID: P60710), Bub3 (ID: Q9WVA3), Xrcc1 (ID: Q60596), and Nek4 (ID: Q9Z1J2) genes; Actin protein (ACTB), Mitotic checkpoint protein (BUB3), DNA repair protein (XRCC1), and Serine/threonine-protein kinase (NEK4) were encoded. Oligonucleotides were initially screened for the absence of dimers or cross-hybridization. Primer pairs with an amplification efficiency of 98–100% were used. qPCR analysis was performed using a Rotor-Gene Q (Qiagen) machine. The detection dye used was 2× SYBR™ Master Mix (Catalog No. K0252, Thermo Scientific™, Lithuania). qPCR was carried out as follows: 95° for 10 min (1 cycle), 95° for 15 s, 58° for 30 s, and 72 °C for 30 s (35 cycles). Identical PCR conditions were created for all genes, and in all cases, results were normalized against Actb used as a housekeeping gene. Relative fold induction expression levels were evaluated with respect to control mice using the standard curve method. The primers used in this study were as follows: for Actb, Fw 5′-TCTGGCACCACACCTTCTAC-3′ and Rv 5′-TTCACGGTTGGCCTTAGGGT-3′; for Bub3, Fw 5′-GTGGCCGGCGCATCG-3′ and Rv 5′-TGGGGCTGAACTTAACCGAG-3′; for Xrcc1, Fw 5′-CTGTATGGCGAGTTCCCTGG-3′ and Rv 5′-CAAAGTTGGGGTCCCACTCC-3′; for Nek4, Fw 5′-CGACTCTATGGCGCGTCTTC-3′ and Rv 5′-GTACTCCATAAACGCGGCCC-3′.

### 2.10. Statistical Analysis

Sample size calculation and power analysis determination were achieved using the G*Power software (ver. 3.1.9.7, Heinrich Heine University, Düsseldorf, Germany). Statistical analysis was performed using Graphpad Prism 6.01 (GraphPad Software Inc., La Jolla, CA, USA). The results are presented as the mean of at least three independent experiments (n ≥ 3) indicating the number of mice used in each section. Statistical differences between groups were determined using unpaired Student’s *t* test. In all cases, differences were considered significant if *p* < 0.05.

## 3. Results

### 3.1. DHEA-Induced PCOS-like Model

**Figure 1:**
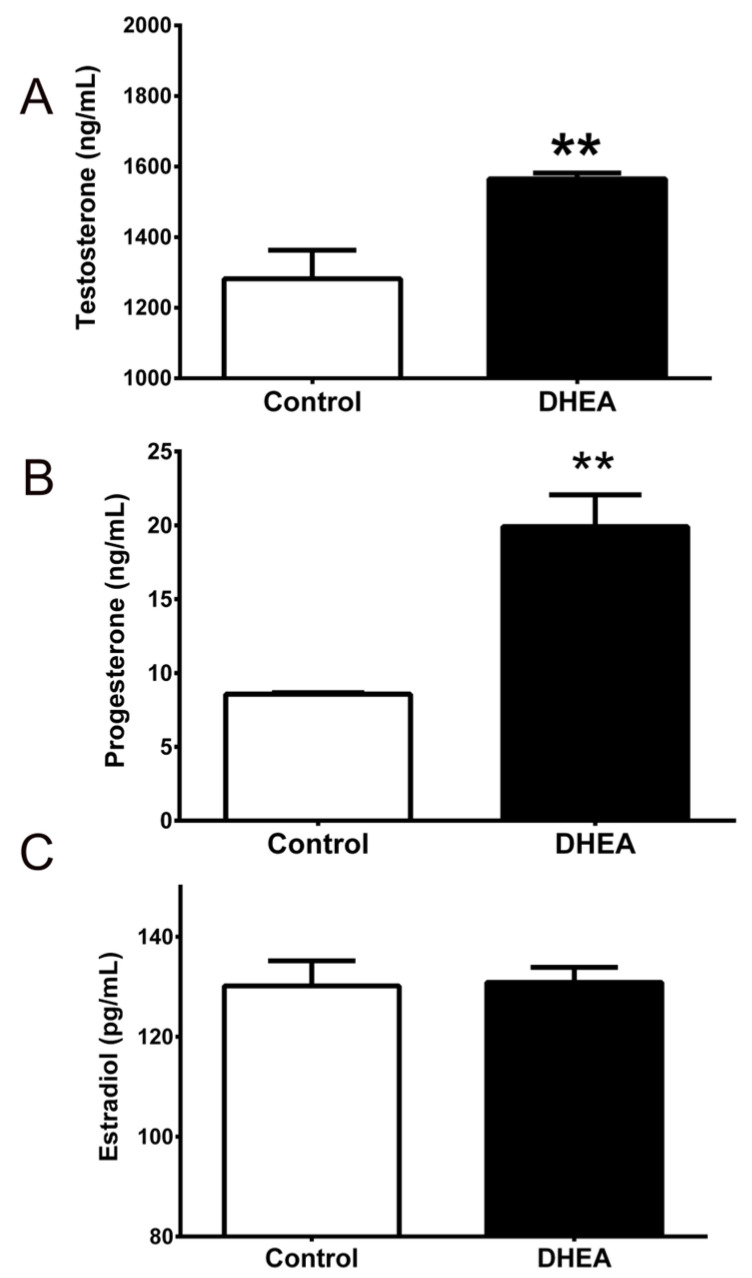
Serum hormone levels in mice with or without DHEA. Serum concentration of (**A**) testosterone, (**B**) progesterone, and (**C**) estradiol in DHEA-treated mice (n = 6) vs. control (n = 6). Each column represents the mean ± SEM. ** *p* < 0.01 compared with the control group.

Morphological, reproductive, and hormonal alterations were evaluated to corroborate PCOS model generation. Mice treated with DHEA lost estrous cyclicity, with metestrus being the prevalent phase. Hormone quantification showed that animals treated with DHEA (n = 6) had higher concentrations of testosterone (1282 ± 81.1 vs. 1565 ± 16.32 ng/dL, *p* = 0.0023) and progesterone (8.56 ± 0.09 vs. 19.91 ± 2.15 ng/mL, *p* = 0.003) in serum as compared with control (n = 6) (Figure 1A,B), while estradiol concentration was not different between groups (130.2 ± 5 vs. 130.9 ± 2.9 pg/mL, *p* = 0.9) (Figure 1C).

**Figure 2:**
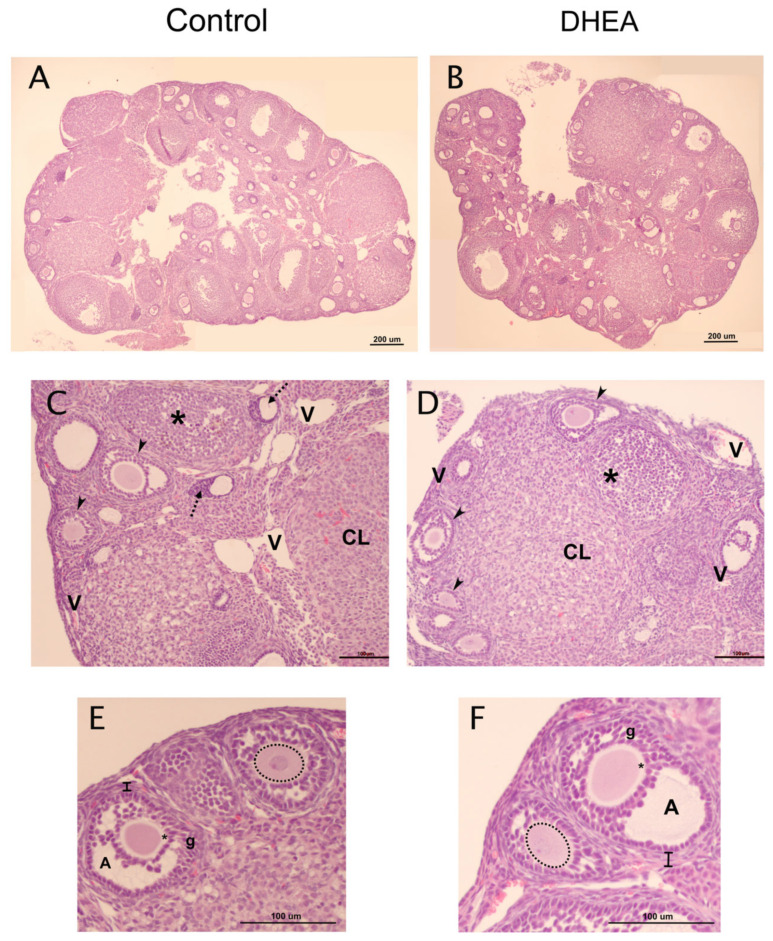
Histological analysis of ovaries of control and DHEA-treated mice. (**A**,**B**) Panoramic view of a control and DHEA-treated mouse ovarian histological section showing follicles in different stages of growth. The antral follicles exhibit this cavity, and other follicles only show granulosa and theca cell layers. (**C**) Ovarian cortex of a control mouse showing some growing atretic follicles with granulosa cells alternated (*) and follicles in advanced atresia (dotted arrow). The boundary between theca and granulosa cells layer is well defined (arrowhead). (**D**) Cortex of the treated mouse ovary showing the boundary between the granulosa and theca cell layers (arrowhead). (**E**) Preantral follicle (right) of control mouse with oocyte exhibiting the nucleus and nucleolus (dotted line). The antral follicle (left) shows the zona pellucida (*) and granulosa cells surrounding the oocyte to form the corona radiata or forming part of the wall. In the theca (line), the presence of capillaries is recognized. (**F**) Detail of the mouse’s ovarian cortical region treated with DHEA shows a preantral follicle (left) with an oocyte, its nucleus, and its nucleolus (dotted line). Antral follicle (right) with well-defined zona pellucida (*) and its ovoid nucleus is visualized. The theca (line) is revealed by the presence of polyhedral cells and capillaries between them. A-F, hematoxylin and eosin staining. Antrum (A); granulosa cells (g); corpus luteum (CL); blood vessels (V).

**Figure 3:**
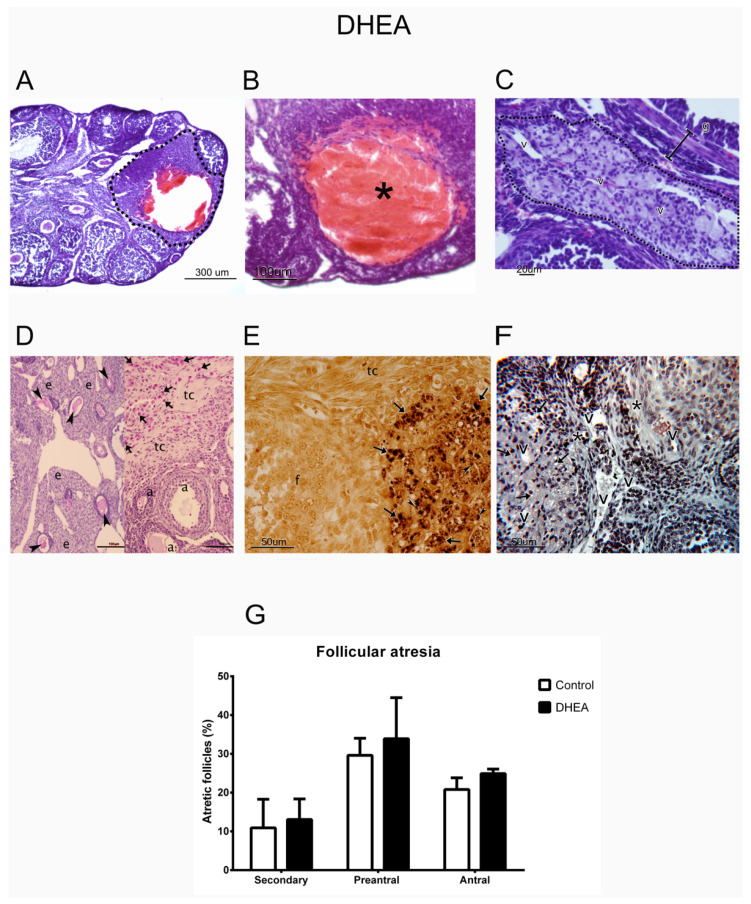
Histological analysis of the ovary of mice treated with DHEA. (**A**) Panoramic view shows the presence of ovarian follicles at different stages of development and a cyst (dotted line). (**B**) Ovarian cyst with a hemorrhagic appearance in the space corresponding to the follicular antrum (*). (**C**) Steroidogenic tissue (dotted lines) shows blood vessels between the cells. (**D**) Atretic follicles surrounded by abundant steroidogenic tissue. Positive PAS reaction in the site that was occupied by oocyte (arrowhead, left) and in cells among the connective tissue (right). (**E**) Set of steroidogenic cells; among these, the presence of apoptotic bodies (arrowhead) and macrophages (arrows) can be observed. Macrophages contain phagocytosed material in their cytoplasm. (**F**) Set of steroidogenic cells (arrows) surrounded by blood vessels of various sizes and connective tissue (*). (**G**) Percentage of atretic follicles in different stages of follicular development in control (n = 3) and DHEA (n = 3). Atretic follicle (a); steroidogenic tissue (e); growing follicle (f); granulosa cells (g); connective tissue (tc); blood vessels (V). Hematoxylin and eosin staining (**A**–**C**); PAS technique (**D**); argentic staining (**E**); Masson’s trichrome stain (**F**).

Morphological analysis showed healthy follicles in both control (n = 3) and DHEA-treated (n = 3) groups and follicles with normal-appearing oocytes and somatic cells (Figure 2). However, some alterations were found in the DHEA group, such as the presence of hemorrhagic cysts (Figure 3A,B), without somatic cell luteinization. In addition, steroidogenic-like tissue was observed in the ovaries of mice treated with DHEA, characterized by polyhedral cells containing lipid droplets and high vascularization (Figure 3C,D). The carbohydrates in the glycoproteins of the zona pellucida of the atretic follicles were identified using the PAS technique (Figure 3E left). Furthermore, follicles were observed in different stages of atresia (Figure 3F right). Silver-impregnated macrophages were observed in the atretic follicles (Figure 3E). Finally, there were a greater number of atretic follicles in animals treated with DHEA (Figure 3G).

### 3.2. DHEA Induces Adverse Pregnancy Outcomes in PCOS-like Model

**Figure 4:**
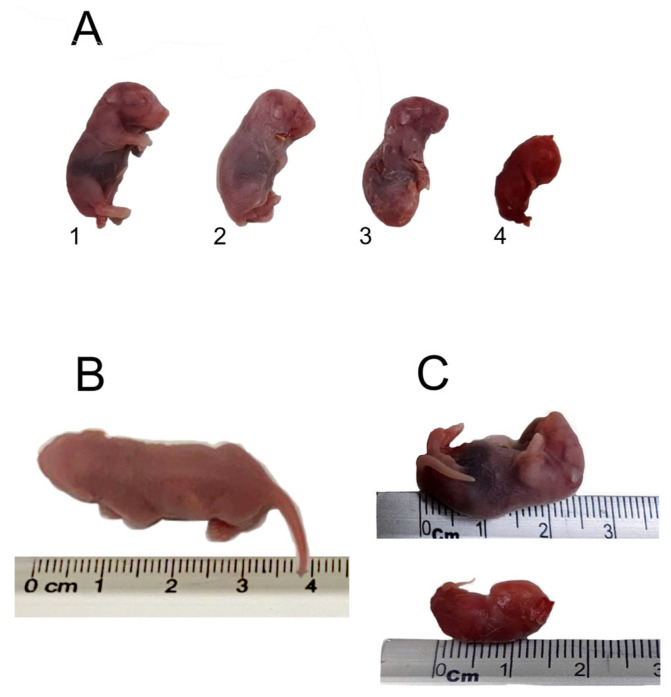
Defects in offspring of mothers treated with DHEA. (**A**) Representative litter of newborns from a female mouse treated with DHEA. (**B**) O-control with standard length and phenotype. (**C**) Malformation in O-DHEA. Offspring of mice treated with DHEA (O-DHEA).

**Figure 5:**
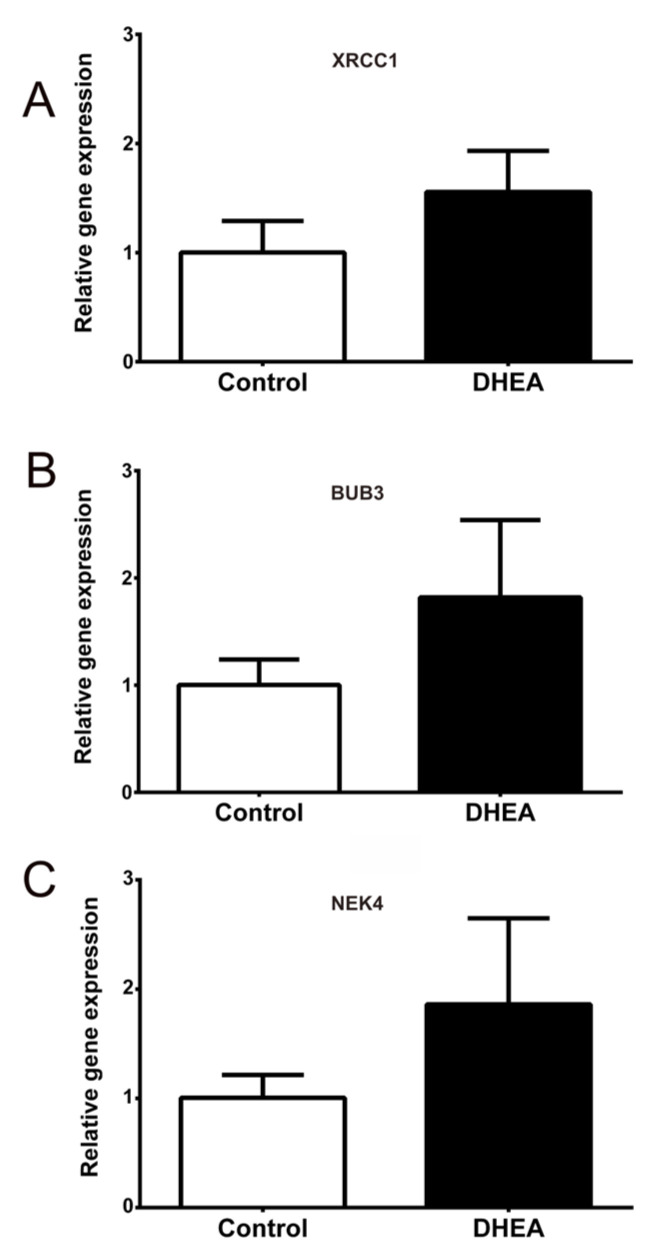
Comparison of Xrcc1, Bub3, and Nek4 gene expression between control (n = 6) and DHEA mice (n = 6). The gene expression of (**A**) Xrcc1, (**B**) Bub3, and (**C**) Nek4 were analyzed by qPCR from ovaries of control (white bars) and DHEA (black bars) mice. The data obtained from the transcripts were normalized against the expression level of the β-actin gene (Actb). Bars indicate the standard deviation (SD).

After treatment, mice were superovulated and mated; however, some pups in the DHEA group had malformed offspring (Figure 4) or died within an hour of delivery. For this reason, we evaluated the expression of genes involved in meiotic cell cycle and DNA repair, which are overexpressed in oocytes of PCOS patients (Xrcc1, Bub3, and Nek4) [21,22]. As expected, the expression of Xrcc1, Bub3, and Nek4 genes in mice that received DHEA (n = 6) was higher than that of the control group (n = 6) (Figure 5).

### 3.3. Maternal Hyperandrogenism Decreases Insulin Sensitivity and Induces Hepatic Gluconeogenesis and Lipid Accumulation

**Figure 6:**
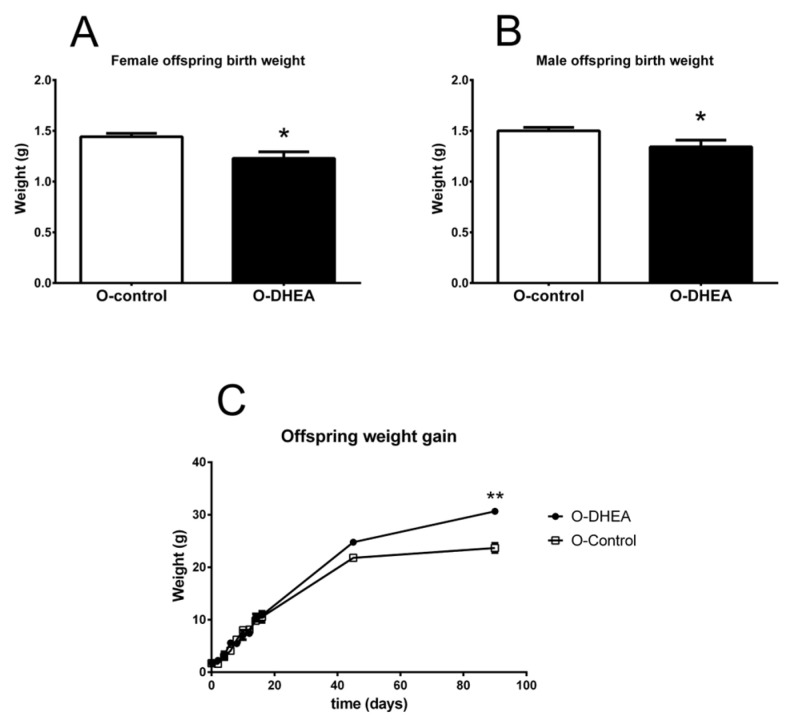
Maternal DHEA treatment increases weight gain in offspring. (**A**) Birth weight in female offspring. (**B**) Birth weight in male offspring. (**C**) Weight gain from 2 to 90 postnatal days of female and male offspring. * *p* < 0.05, ** *p* < 0.001 compared with O-control. Values are means ± SEM. O-control female, n = 10; O-control male, n = 8; O-DHEA female, n = 6; O-DHEA, male n = 7). Offspring of mice treated with DHEA (O-DHEA); Offspring of control mice (O-control).

Both O-control and O-DHEA pups were weaned at 30 days, from which moment they had free access to food and water under controlled light and temperature conditions in the vivarium. The weight and food consumption of the offspring in both groups were recorded for three months. Once weaned, the food consumption of all mice was similar, regardless of the group to which they belonged (data not shown). O-DHEA males (n = 7) and females (n = 6) were born with a lower weight than the offspring in the control group (n = 10 females, 8 males) (males 1.5 ± 0.03 vs. 1.34 ± 0.06 g, *p* = 0.02; female 1.44 ± 0.03 vs. 1.22 ± 0.06, *p* = 0.01) (Figure 6A,B). Mice in the O-DHEA group gained more weight than those in the control group as time went by, which became evident 40 days after birth and showed a significant difference at 90 days (23.67 ± 0.88 vs. 30.67 ± 0.33 g, *p* = 0.0018) (Figure 6C).

**Figure 7:**
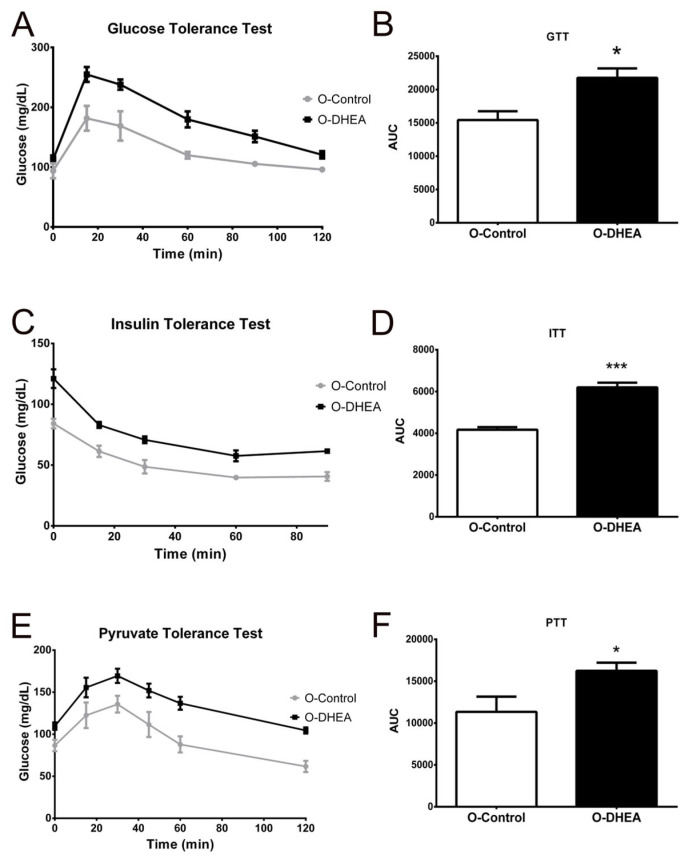
DHEA induces metabolic changes in 4-month-old offspring. (**A**) Blood glucose levels during intraperitoneal glucose tolerance test upon 12 h of fasting. (**B**) Area under the glucose tolerance test curve. (**C**) Blood glucose levels during intraperitoneal insulin tolerance test upon six hours of fasting. (**D**) Area under the curve of insulin tolerance test. (**E**) Blood glucose levels during intraperitoneal pyruvate tolerance test upon 6 h of fasting. (**F**) The area under the curve of the pyruvate tolerance test. * *p* < 0.05, *** *p* < 0.0001 compared with O-control. Values are means ± SEM. O-control, n = 18; O-DHEA, n = 13. Area under the curve (AUC); glucose tolerance test (GTT); insulin tolerance test (ITT); offspring of mice treated with DHEA (O-DHEA); offspring of control mice (O-control); pyruvate tolerance test (PTT).

Because PCOS is strongly related to metabolic diseases [25], beginning in childhood and puberty of patients [26], we decided to analyze some metabolic parameters in 3-month-old pups. First, we evaluated fasting blood glucose level. We found that O-DHEA (n = 13) had higher glucose concentration after 6 h of fasting as compared to O-control (n = 18) (91.95 ± 2.93 vs. 112.2 ± 19.67 mg/dL, *p* = 0.0003). Subsequently, we performed GTT on the pups in both groups. Figure 7A shows that after the intraperitoneal administration of the glucose load, O-DHEA mice present a higher glucose concentration over time; after 120 min, the values do not reach basal levels. Moreover, the area under the GTT curve (AUC) is significantly higher in the O-DHEA group (Figure 7B) (15,428 ± 1318 vs. 21,763 ± 1415 (mg/dL × min), *p* = 0.02). 

Subsequently, ITT was performed to assess insulin sensitivity. As shown in Figure 7C, the O-DHEA group (n = 13) had higher glucose concentrations throughout the experiment, so the AUC is significantly higher than the O-control group (n = 18) (Figure 7D) (4169 ± 125 vs. 6197 ± 226 (mg/dL × min), *p* = 0.0007). Finally, in order to evaluate hepatic gluconeogenesis, PTT was performed, showing that O-DHEA mice (n = 13) after intraperitoneal pyruvate administration had higher glucose production than O-control mice (n = 18) (Figure 7E). The AUC corresponding to PTT was higher in O-DHEA (11,339 ± 1825 vs. 16,242 ± 978 (mg/dL × min), *p* = 0.03) (Figure 7F), indicating that enhanced gluconeogenesis and insulin resistance resulted in higher fasting glucose levels.

**Figure 8:**
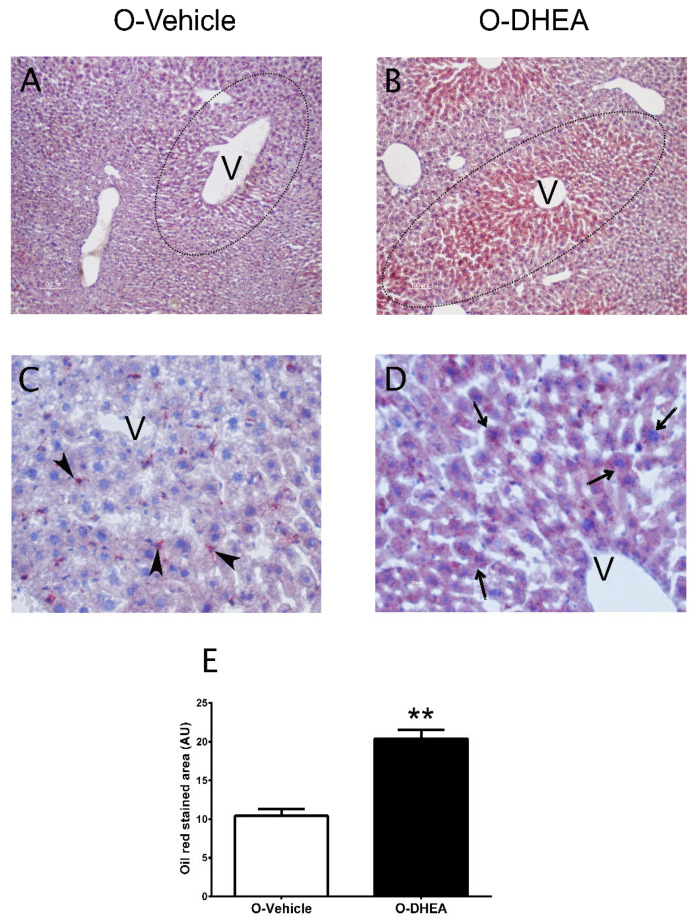
Neutral lipids stained by Oil Red O in liver sections from O-control and O-DHEA mice. (**A**,**B**) Liver sections show the organization of hepatocyte cords (dotted line) around the central vein (V) and neutral lipid accumulation in the cytoplasm of hepatocytes of the O-DHEA group. (**C**,**D**) Detail of the hepatic tissue cords showing the central vein of a hepatic lobule and the sinusoids (s). (**C**) shows the presence of Ito cells (arrowhead), also called perisinusoidal cells. Ito cells show that the positive reaction to the Oil Red O staining is due to the capacity to store lipids, which is why they are called lipocytes. In the case of (**D**), predominantly hepatocytes accumulate lipids in their cytoplasm. (**E**) Quantification of Oil Red O-stained area. ** *p* < 0.001 compared with O-control. Values are means ± SEM. O-control, n = 18; O-DHEA, n = 13. Arbitrary units (AU); offspring of mice treated with DHEA (O-DHEA); offspring of control mice (O-control); central vein (V).

Cholesterol and triacylglycerol levels were quantified after fasting to assess whether metabolic changes were also reflected in both parameters. The results showed similar concentrations of cholesterol and triacylglycerols in both groups (data not shown). However, Oil Red O staining of liver sections and their quantification showed higher hepatic accumulation of neutral lipids in the periphery of the central vein of the O-DHEA group (n = 13) compared with O-control mice (n = 18) (10.42 ± 0.88 vs. 20.39 ± 1.15, *p* = 0.001) (Figure 8A–E), suggesting predisposition to fatty liver disease.

## 4. Discussion

Despite having a strong genetic component, polycystic ovary syndrome (PCOS) is a reproductive and endocrine–metabolic disorder of unknown etiology. Currently, the induction of PCOS in mice through the administration of DHEA is a widely used model. In this work, we observed that DHEA administration modified the follicular microenvironment, thereby damaging the oocyte. In addition, as occurs in women with PCOS [27], the serum testosterone and progesterone levels were elevated in this biologic model, without changes in estradiol levels. Women with PCOS present a decrease in serum estrogen levels attributed to the increase in AMH that inhibits placental aromatase (CYP19A1), which correlates negatively with FSH, a gonadotropin responsible for estrogen biosynthesis [28].

Maternal HA has been proposed as the leading cause of the transmission of metabolic and reproductive disorders [29]. It has been found that daughters of PCOS patients have a 5-fold higher risk of presenting PCOS than normal patients [29]. Moreover, it has been shown that daughters and sons of PCOS patients have an increased risk of metabolic and behavioral alterations, including lower APGAR scores in infants, delayed development and early growth, obesity, and T2D [14,15,30].

Offspring of female mice treated with DHEA and mated with control males exhibited malformations or low weight. It has been reported that 30 to 50% of women with PCOS experience early abortions and are at increased risk of pregnancy and perinatal complications [31]. Furthermore, a high rate of embryo implantation failure has been detected in mice treated with DHEA, suggesting that the intrauterine environment is altered [32].

Considering that the ovarian morphology of both the control mothers and the DHEA group was similar, it is assumed that the oocyte suffers intrinsic defects caused by androgen excess. In line with Wood et al. [22], this study revealed an increase in the expression of Bub3 and Nek4 in mice treated with DHEA. BUB3 is part of the spindle assembly checkpoint (SAC), which is activated by DNA damage, arresting the oocyte in the metaphase of meiosis I (MI) [33]. In addition, SAC is responsible for reviewing the union of the kinetochores to the spindle microtubules to avoid errors in chromosome segregation [34]. In humans, the first cause of pregnancy failure and congenital diseases is errors in chromosome segregation in oocytes. Therefore, we assumed that the increase in expression of Bub3 in mice treated with DHEA was indicative of DNA damage and possible chromosome segregation failures, resulting in miscarriages and offspring malformations. In fact, Wood et al. [22] found in oocytes from PCOS women that Bub3 increases its expression 2.3-fold, which is associated with failure in embryonic development. Meanwhile, NIMA-related kinases (NEK) consist of 11 family members involved in regulating the cell cycle, meiosis, and the response to DNA damage, among others [35]. Nek4 is overexpressed in oocytes from PCOS patients, which could also be a biomarker in this pathology to abnormalities in early embryonic development [22].

On the other hand, one of the main factors of DNA damage is oxidative stress, which is strongly related to the pathogenesis of several diseases, including PCOS and its associated alterations, such as IR, obesity, and HA [36]. XRCC1 is a protein involved in several DNA repair pathways during oxidative stress [37,38]. Single-cell oocyte transcriptome has shown that Xrcc1 is overexpressed and more active in oocytes from patients with PCOS, indicating DNA damage in these early stages of oocyte maturation [21], as proposed in this work.

This study revealed that miscarriages, offspring with malformations, and low birth weight were more frequent in mice treated with DHEA. Maternal and environmental factors, such as a high-fat diet, have adverse effects on offspring, mainly when exposure occurs in preconception, which means that fetal programming depends on the environment intrauterine, even before implantation [39,40]. Therefore, increase in the expression of Bub3, Xrcc1, and Nek4 by DHEA could be due to changes in DNA, affecting the gene expression in the oocyte by DHEA treatment, which in turn generates defects in embryonic development.

Intrauterine growth retardation is associated with development of metabolic diseases in adult life [41]. IR, T2D, and cardiovascular diseases are associated with low birth weight [42]. In human studies and animal models, excess prenatal androgens have been found to induce delayed fetal growth and low birth weight [43,44]. Therefore, the low birth weight in O-DHEA observed in this work supports alterations in the intrauterine environment. Moreover, Sir-Petermana et al. [45] found a higher prevalence of small gestational age in newborns of PCOS patients. Furthermore, Ibáñez et al. [46] proposed low birth weight as a predictor of hyperinsulinemia and ovarian HA in girls. Androgen excess during pregnancy has also been shown to favor postnatal catch-up growth [43,44]. Children who gain weight rapidly during the first years of childhood are fatter and have more abdominal fat, increasing the risk of developing metabolic diseases in adulthood [47,48,49,50]. In addition, children of mothers with PCOS have a higher body mass index in early infancy and childhood [15,18]. The risk of developing metabolic diseases is increased in the presence of IR, and the concentration of insulin, triacylglycerols, and cholesterol is elevated before puberty and in adult life in the children of hyperandrogenic PCOS women [18,51,52].

Our results showed that the O-DHEA group had a higher blood glucose concentration after a 6 h fast and in the GTT and the ITT, regardless of gender. These results indicated a reduced ability to metabolize glucose, IR, impaired beta cell function, and other alterations related to carbohydrate metabolism. In fact, prenatally androgenized mice, increased fasting glucose, and impaired glucose tolerance have been shown, but without changes in insulin sensitivity [53]. However, in other animal models, exposure to excess testosterone during fetal development generated IR in females [54]. Furthermore, prenatal exposure to testosterone affects the ability of the pancreas to secrete insulin and predisposes IR development [55]. Moreover, according to Noroozzadeh et al. [56], maternal HA increases the risk of developing T2D in the daughters of PCOS mothers because they have fasting glucose ≥ 126 mg/dL or 2 h glucose ≥ 200 mg/dL. Although, according to fetal programming, lifelong health diseases have their origin in fetal life [41], our results support that maternal HA decreases insulin sensitivity, favoring the development of T2D in the offspring.

The glucose values obtained in the PTT showed that the O-DHEA group had higher hepatic glucose production, thus contributing to fasting hyperglycemia. As has already been shown in a PCOS rat model generated by the administration of testosterone propionate and a high-fat diet, increased hepatic gluconeogenesis reveals an early glucose and lipid metabolism failure [57]. In addition, the administration of dihydrotestosterone (DHT) induces the transcription of gluconeogenic enzymes and the binding of androgen receptors to phosphatidylinositol-3-kinase (PI3K)-p85, increasing gluconeogenesis and decreasing insulin action [58]. Furthermore, in females treated with DHT, hepatic androgen receptor deletion prevented both IR and gluconeogenesis, supporting the role of androgens in metabolic disturbances [59]. In this work, we show that these metabolic alterations are present in male and female pups of hyperandrogenic female mice.

PCOS is a risk factor for the development of steatosis and liver damage in patients with IR and HA [60]. Moreover, hepatic lipid accumulation is strongly related to the development of other comorbidities. For this reason, we consider that gluconeogenesis in the O-DHEA group uses glycerol from the degradation of triacylglycerols as a substrate as a compensatory IR mechanism. Our results demonstrated a higher amount of neutral lipids in the liver of the O-DHEA group. HA, IR, and obesity, which are present in a large percentage of patients with PCOS, are factors for the generation of nonalcoholic fatty liver disease (NAFLD) in offspring and with a high probability of progressing to nonalcoholic steatohepatitis (NASH). In animal models, fetal androgen excess is associated with the development of a cholestasis-like condition, accompanied by hypercholesterolemia and hyperinsulinemia, as well as markers of liver fibrosis [61]. Previously, female pups of DHEA-treated mice were shown to overexpress glycolipid metabolism genes [62]. We show that males and females present alterations in the hepatic accumulation of lipids.

Maternal obesity, caused by a high-fat diet, has been shown to have a transgenerational impact on the generation of fibrosis, inflammation, and hepatic steatosis, in addition to changes in the composition of bile acids in adult life, especially when offspring follow a diet rich in lipids and carbohydrates [63]. Mothers with high-fat diet and IR results in a more significant hepatic accumulation of triacylglycerols, increased de novo lipid biosynthesis, and inflammation in offspring [64]. Moreover, maternal metabolic syndrome is associated with susceptibility to the development of fatty liver in mouse pups due to changes in the expression of genes involved in regulating hepatic lipid metabolism [65].

## 5. Conclusions

In conclusion, maternal hyperandrogenism induces changes in oocytes, which impair the metabolism of offspring, manifested by insulin resistance, increased gluconeogenesis, and liver fat accumulation, thereby increasing the risk of DT2 among other chronic diseases in adulthood.

## Data Availability

Not applicable.
